# An epidemiologic survey of road traffic accidents in Iran-2017 

**Published:** 2019-08

**Authors:** Hossein Akbari, Malihe MohammadZade, Fatemeh Sadat Asgarian, Mehrdad Mahdian

**Affiliations:** ^*a*^Social Determinants Of Health (SDH) Research Center, Kashan University of Medical Sciences, Kashan, Iran.; ^*b*^Kashan University of Medical Sciences, Kashan, Iran.; ^*c*^Trauma Research center, Kashan University of Medical Sciences, Kashan, Iran.

## Abstract

**Background:**

Accidents have always been recognized as one of the most important causes of disability or death in humans. The purpose of this study, determining the frequency of causes of accidents and incidents in Iran in 1395 to provide further prevention.

**Methods:**

This is a cross-sectional study where the data recorded accidents and incidents in the country have been used in 1395. This information was extracted according to age, sex and type of incident, and was reported by frequency and percentage.

**Results:**

This year, there were 1489862 incidents in the country that about 446,238 were women (29.9%). The most frequent causes was of the car accidents (15.5%), fall from the high (13.7%) And motorcycle accidents (10.2 percent). The largest incident was at home with a frequency of 36% and then there was Alley and street (32%).

**Conclusions:**

The results of this study, the frequency of accidents and incidents was higher in the men and Age group 25-15 years that requires more attention from health policy makers.

**Keywords:**

Accident, Survey, Iran

